# Aβ modulates actin cytoskeleton via SHIP2-mediated phosphoinositide metabolism

**DOI:** 10.1038/s41598-019-51914-2

**Published:** 2019-10-29

**Authors:** Hae Nim Lee, Kyoung Mi Sim, Hyunbin Kim, Jeongmin Ju, Ae Nim Pae, Jae-Bong Park, Hoon Ryu, Jihye Seong

**Affiliations:** 1 Convergence Research Center for Diagnosis Treatment Care of Dementia, Korea Institute of Science and Technology, Seoul, 02792 Republic of Korea; 2 Department of Integrated Biomedical and Life Science, Korea University, Seoul, 02708 Republic of Korea; 3 Department of Converging Science and Technology, Kyung Hee University, Seoul, 02453 Republic of Korea; 4 Division of Bio-Medical Science & Technology, KIST School, Korea University of Science and Technology, Seoul, 02792 Republic of Korea; 5 Department of Biochemistry, Hallym University College of Medicine, Chuncheon, 24252 Republic of Korea; 6 Center for Neuroscience, Korea Institute of Science and Technology, Seoul, 02792 Republic of Korea

**Keywords:** Phosphoinositol signalling, Mechanisms of disease

## Abstract

Emerging evidences suggest that phospholipid metabolism is altered in Alzheimer’s disease (AD), but molecular mechanisms on how this affects neurodegeneration in AD is poorly understood. SHIP2 is a phosphoinositide-metabolizing enzyme, which dephosphorylates PI(3,4,5)P_3_ resulting to PI(3,4)P_2_, and it has been recently shown that Aβ directly increases the activity of SHIP2. Here we monitored, utilizing fluorescent SHIP2 biosensor, real-time increase of PI(3,4)P_2_-containing vesicles in HT22 cells treated with Aβ. Interestingly, PI(3,4)P_2_ is accumulated at late endosomes and lysosomal vesicles. We further discovered that ARAP3 can be attracted to PI(3,4)P_2_-positive mature endosomes via its PH domain and this facilitates the degradation of ARAP3. The reduced level of ARAP3 then causes RhoA hyperactivation and filamentous actin, which are critical for neurodegeneration in AD. These results provide a novel molecular link between Aβ and actin disruption through dysregulated phosphoinositide metabolism, and the SHIP2-PI(3,4)P_2_-ARAP3-RhoA signaling pathway can be considered as new therapeutic targets for synaptic dysfunctions in Alzheimer’s disease.

## Introduction

Phosphoinositides (PIs) play crucial roles in dynamic cellular processes such as membrane trafficking, thus spatiotemporal distribution of different phosphoinositides should be tightly controlled by their metabolizing enzymes^1–3^. For example, at the initial stage of clathrin-mediated endocytosis (CME), PI(3,4)P_2_ at plasma membrane binds to clathrin adaptor protein AP-2 which is crucial for the formation of endocytic vesicle coats^4,5^. After this nucleation stage, synaptojanin-p170 can be recruited by clathrin-coat components and then dephosphorylates PI(4,5)P_2_ into PI4P^6,7^. PI4P can be further phosphorylated by PI3K C2α and then produce PI(3,4)P_2_^2,3,8^.

PI(3,4)P_2_ can be also generated from PI(3,4,5)P_3_ by Src homology domain-containing inositol 5-phosphatase 2 (SHIP2), which is recruited to clathrin coated pits (CCPs) via intersectin^9,10^. PI(3,4)P_2_ then recruits a BAR domain-containing protein SNX9^2^, which allows the shape of CCPs to be competent for fission by dynamin^11,12^. In addition to its role in CME, PI(3,4)P_2_ is also suggested to be crucial for fast endophilin-mediated endocytosis (FEME), a clathrin-independent endocytic pathway^13^. During FEME, SHIP2 activity is important to generate PI(3,4)P_2_ which then recruits endophilin, a BAR domain-containing protein^13,14^. After the fission of endocytic vesicles, PI(3,4)P_2_ is suggested to be dephosphorylated by inositol-3, 4-bisphosphate 4-phosphatse (INPP4), and the resulting PI3P is known as a major component of early endosomes^15^. During endosome maturation process, PI3P is phosphorylated by PIKfyve producing PI(3,5)P_2_, a major phospholipid in late endosomes and lysosomes^1,16^. Therefore, spatiotemporal distribution of phosphoinositides are tightly controlled during dynamic endocytosis and endosome-lysosome maturation process.

Emerging evidences suggest that phospholipid metabolism is altered in AD^17–20^. For example, a recent study reported that oligomeric amyloid β (Aβ) activates SHIP2 via FcγRIIb to increase the level of PI(3,4)P_2_, which then causes tau hyperphosphorylation in AD^21^. SHIP2 is composed of an N-terminal SH2 domain, a catalytic domain, a C2 domain, an NPXY motif, proline-rich regions, and a C-terminal sterile α motif (SAM) domain^22^. SHIP2 can be recruited to plasma membrane by the interaction of its SH2 domain and the phosphorylated tyrosine of the activated receptors such as IGF-1R and FcγRIIb^21,23,24^. At the plasma membrane, the catalytic domain of SHIP2 can function as an inositol 5-phosphatase for PI(3,4,5)P_3_ producing PI(3,4)P_2_, and the following C2 domain has been recently suggested to enhance the catalytic turnover^22^. Other domains of SHIP2 mediate interactions with various signaling proteins, for example, Shc, Abl, filamin, intersectin, ARAP3, and EphA2^10,25–29^, thus SHIP2 can be involved in many cellular processes such as cell adhesion, spreading, receptor endocytosis, and insulin signaling^29–31^.

In particular, SHIP2 directly binds to ARAP3, ArfGAP with RhoGAP domain, Ankyrin repeat and PH domain 3, through the interaction of its C-terminal SAM domain with the SAM domain of ARAP3^28,32^. ARAP3 is a GTPase activating protein (GAP) for Arf6 and RhoA, which contains an N-terminal SAM domain, 5 PH domains and a Ras-binding domain (RBD)^33^. It has been well known that ARAP3 is a negative regulator for RhoA^33,34^, which can be further related to neuronal actin cytoskeleton. Interestingly, the function of ARAP3 is also tightly regulated by phosphoinositide metabolism, as ARAP3 can be recruited to plasma membrane where it functions, through the interaction with PI(3,4,5)P_3_ via its N-terminal PH domains^35^. While the phosphoinositide metabolism is dysregulated in AD^19,21^, it is not clear yet whether this altered phosphoinositide metabolism also affects the function of ARAP3 and contributes to the neurotoxicity in Aβ-induced AD model. In fact, hyperactivation of RhoA and the accumulation of filamentous actin (F-actin) have been reported in AD^36^, and ARAP3 may be involved in AD as a negative regulator for RhoA^33,34^. Thus, we decided to investigate how Aβ-induced alteration of phosphoinositide metabolism via SHIP2 activation affects the function of ARAP3, actin cytoskeleton and neurotoxicity.

To monitor the real-time SHIP2 activity and dynamic spatiotemporal distribution of PI(3,4)P_2_ in live cells, a fluorescent SHIP2 biosensor was generated by fusing a TAPP1-PH domain, which specifically binds to PI(3,4)P_2_, and a red fluorescent protein mKate2^37,38^. In addition, RhoA activity in live cells was measured by a RhoA biosensor based on fluorescence resonance energy transfer (FRET)^39^. We also applied bimolecular fluorescence complementation (BiFC) technique to identify ARAP3 PH domain capable of binding to PI(3,4)P_2_. Utilizing these fluorescent biosensors, we discovered that Aβ increases the SHIP2-mediated production of PI(3,4)P_2_, particularly at late endosomes and lysosomal vesicles. This accumulation of mature endosomes containing PI(3,4)P_2_, which can directly interact with the PH domain of ARAP3, facilitates the degradation of ARAP3 through lysosomal pathway. We further showed that the reduced ARAP3 level can cause the hyperactivation of RhoA and subsequent formation of F-actin. These results provide a novel molecular link between Aβ and actin disruption through dysregulated phosphoinositide metabolism. As actin disruption can further contribute to degeneration of neurites and synaptic dysfunctions in AD^36,40,41^, the Aβ-SHIP2-ARAP3-RhoA signaling pathway can be considered as new therapeutic candidates for Alzheimer’s disease.

## Results

### Aβ induces the SHIP2-mediated PI(3,4)P_2_ accumulation at vesicles

**Figure 1:**
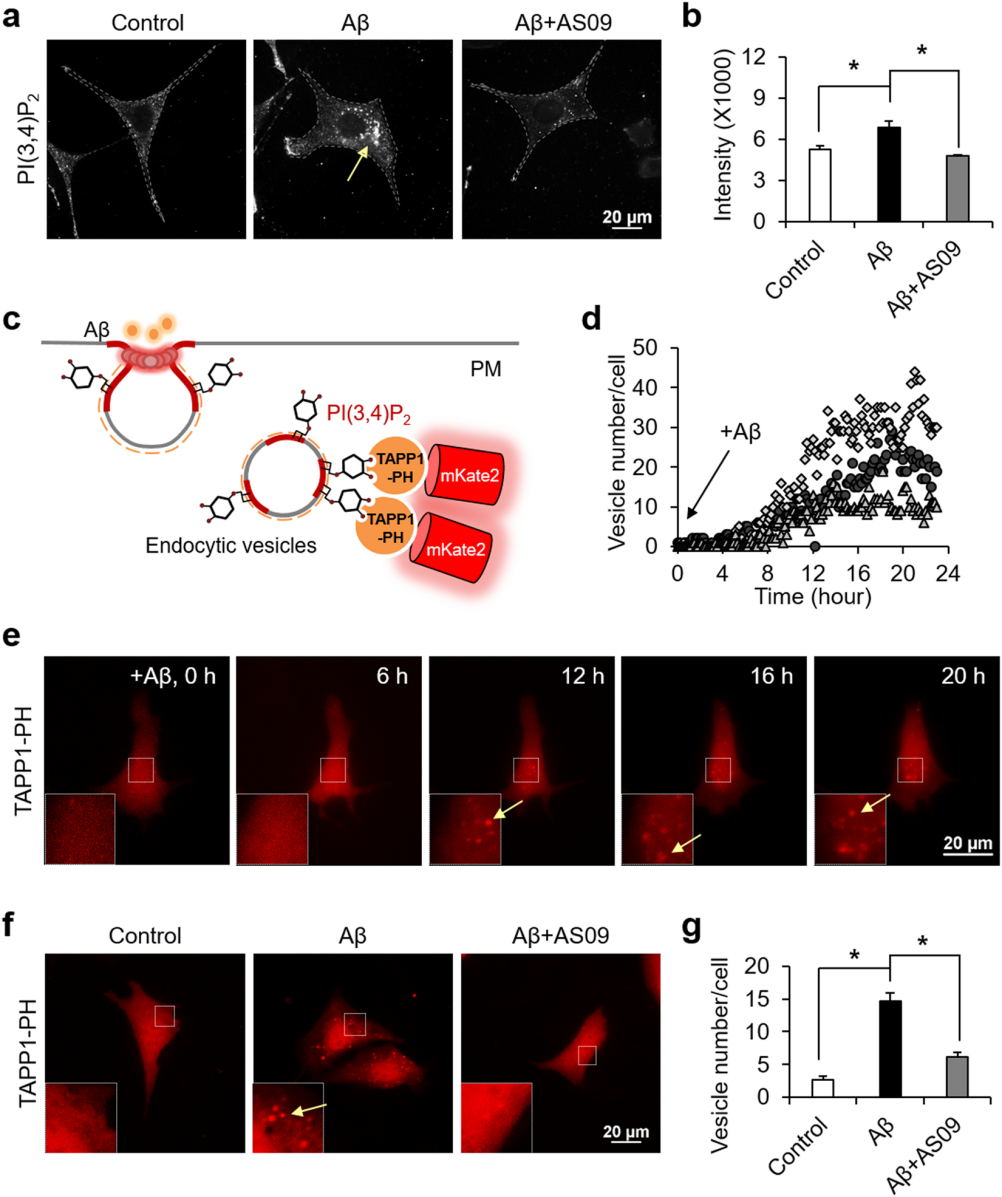
Aβ increases SHIP2-mediated PI(3,4)P2 accumulation at vesicles. (**a**,**b**) Representative images of PI(3,4)P_2_ in HT22 cells stimulated with 1 μM Aβ in the absence or presence of 10 μM AS09 for 24 hr (**a**) and the quantification of fluorescence intensity (**b**) are presented (means ± SEM; *t*-test; *p < 0.05; *n* = 48~55 cells per group). (**c**) Schematic illustration of mKate2-TAPP1-PH sensor to visualize the distribution of PI(3,4)P_2_-containing vesicles. (**d**,**e**) Time-lapse analysis (**d**) and representative images (**e**) of PI(3,4)P_2_-containing vesicles in HT22 cells after the treatment of 1 μM oligomeric Aβ for 24 hr (*n* = 3). Insets show the boxed areas at high magnification and yellow arrows indicate PI(3,4)P_2_-containing vesicles. (**f**,**g**) Representative images of mKate2-TAPP1-PH in HT22 cells stimulated with 1 μM Aβ in the absence or presence of 10 μM AS1938909 (AS09) for 24 hr (**f**) and the quantification of vesicle numbers per each cell (**g**) are presented (means ± SEM; *t*-test; *p < 0.05; *n* = 41~46 cells per group).

It has been shown that Aβ can induce the activity of SHIP2 and increase the level of PI(3,4)P_2_^21^. For the detection of subcellular distribution of PI(3,4)P_2_ induced by 1 μM of oligomeric Aβ, we performed the immunocytochemistry staining and found the perinuclear accumulation of PI(3,4)P_2_-containing vesicles (Fig. 1a,b). This Aβ-induced increase of PI(3,4)P_2_ vesicles was prevented by SHIP2 inhibitor AS1938909 (AS09) (Fig. 1a,b, Supplementary Fig. S1a) or SHIP2 siRNA (Supplementary Fig. S1b,c). These data indicate that Aβ increases the SHIP2-mediated accumulation of PI(3,4)P_2_-positive vesicles near perinuclear regions. Oligomeric Aβ was prepared as previously described^42^, and its toxicity was confirmed with WST colorimetric assay in mouse hippocampal neuronal HT22 cells and human neuroblastoma SHSY5Y cells. When these cells were treated with 1 μM of oligomeric Aβ for 24 hr, cell viability was significantly reduced down to around 60% (Supplementary Fig. S2a,b). While its activity is increased, total expression level of SHIP2 was not changed by the incubation of Aβ for 24 hr in HT22 cells and SHSY5Y cells (Supplementary Fig. S2c,d).

To investigate the real-time SHIP2 activity in live cells with high spatiotemporal resolutions, we generated a fluorescent protein-based SHIP2 sensor containing a PH domain of TAPP1 and red fluorescent protein mKate2 (Fig. 1c). As the TAPP1-PH domain specifically binds to PI(3,4)P_2_, a product of SHIP2 activity^37,38^, this red-colored fluorescent sensor can visualize dynamic change of PI(3,4)P_2_ distribution induced by SHIP2 in live cells. In fact, we can observe real-time increase of PI(3,4)P_2_ at vesicles near perinuclear regions in response to 1 μM of Aβ oligomers (Fig. 1d,e; Supplementary Video 1). These vesicles started to appear after around 10 hr of Aβ incubation, and the number of vesicles was gradually increased until around 20 hr (Fig. 1d,e). The number of PI(3,4)P_2_-containing vesicles was significantly higher in the Aβ-treated group comparing to negative control, and this Aβ-induced increase of PI(3,4)P_2_ vesicles was prevented by SHIP2 inhibitor, AS09 (Fig. 1f,g), suggesting that the increase of PI(3,4)P_2_-containing vesicles is induced by Aβ-mediated SHIP2 activation.

### ARAP3 is physically associated with PI(3,4)P2-containing vesicles via its PH domains

**Figure 2:**
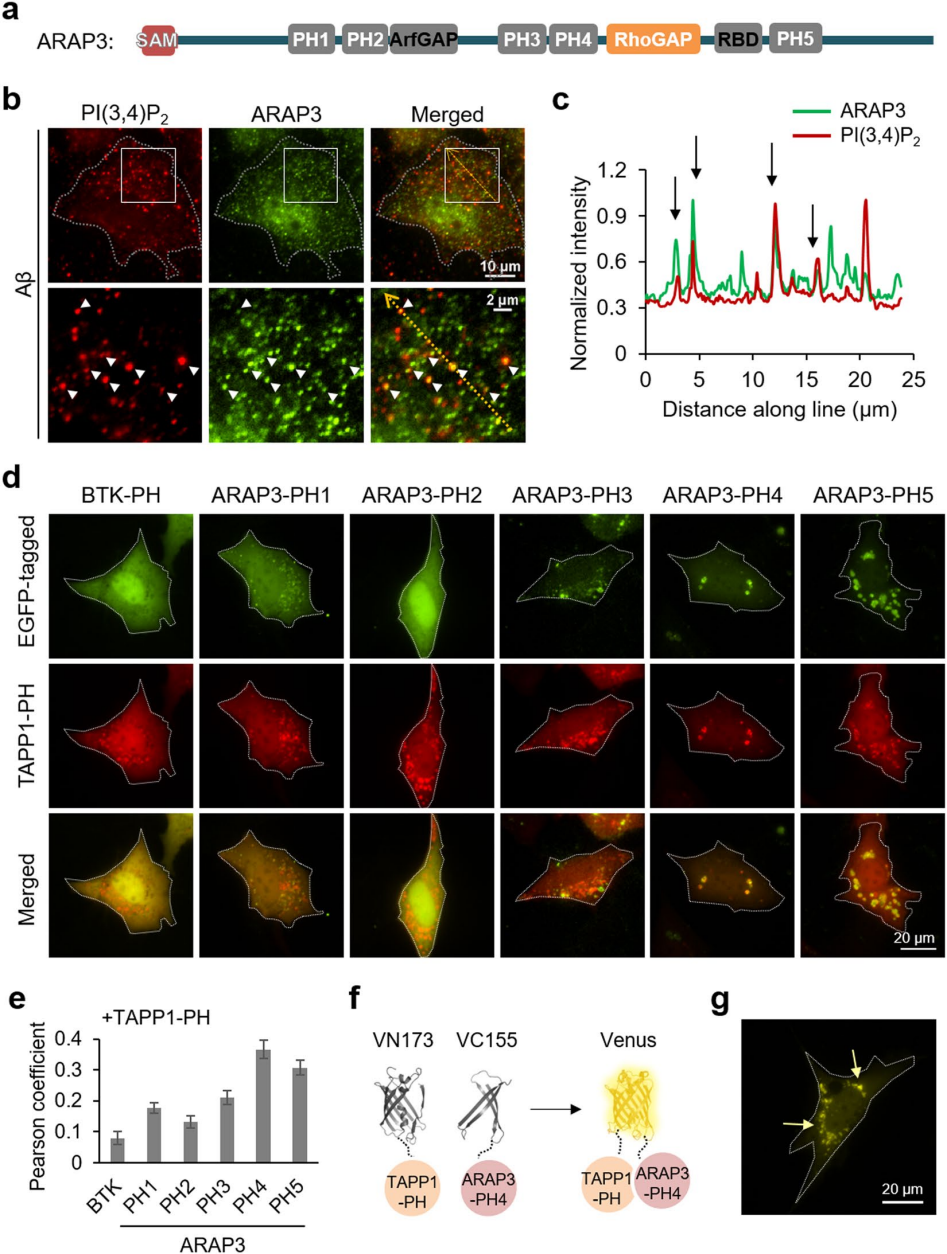
ARAP3 can be associated with PI(3,4)P2-containing vesicles via its PH4 domain. (**a**) The scheme of ARAP3 domains. (**b**) ARAP3 (green) is partially localized at PI(3,4)P_2_-containing vesicles (red) in HT22 cells treated with 1 μM of Aβ for 24 hr. Lower panels show the boxed areas of upper panels at high magnification. (**c**) Intensity profiles of PI(3,4)P_2_ and ARAP3 across the yellow line in the merged image shown in (**b**). (**d**) EGFP-tagged each PH domain of ARAP3 (green), mKate2-TAPP1 (red) and merged images of HT22 cells after 48 hr starvation. Btk PH domain-EGFP was used as control. (**e**) Pearson coefficient was calculated to measure colocalization between TAPP1-PH and each ARAP3-PH construct (n = 16, 61, 64, 34, 49, 30 for Btk PH, ARAP3 PH1, PH2, PH3, PH4 and PH5, respectively) (**f**,**g**) BiFC design of VN173-TAPP1 PH domain and VC-155-ARAP3 PH4 domain (**f**) and BiFC signal in HT22 cells after 48 hr starvation (**g**). Yellow arrows highlight the BiFC signal at vesicles.

We next investigated the effect of the SHIP2-induced production of PI(3,4)P_2_-containing vesicles. It has been shown that SHIP2 can directly bind to ARAP3 through their SAM domain interaction^28^. In addition, ARAP3 contains 5 PH domains (PH1, PH2, PH3, PH4, PH5) (Fig. 2a), which can potentially interact with phosphoinositides^43^. In fact, it has been shown that ARAP3 can bind to PI(3,4,5)P_3_ via its N-terminal PH domains PH1 and PH2^35^, but whether ARAP3 can directly interact with PI(3,4)P_2_ is not clear. Thus, we check the distribution of ARAP3 after 24 hr of Aβ treatment, and interestingly, we can observe that ARAP3 is partly located at the PI(3,4)P_2_-containing vesicles (Fig. 2b,c).

To further identify which PH domain of ARAP3 is capable of binding to PI(3,4)P_2_, we generated constructs containing EGFP and each PH domain of ARAP3^35^ (Supplementary Fig. S3), and check whether each EGFP signal can be colocalized with mKate2-tagged TAPP1 PH domain. Our results show that ARAP3 PH4 and PH5 domains can be colocalized with PI(3,4)P_2_-containing vesicles (Fig. 2d,e, Supplementary Fig. S4). PH4 domain displayed the highest correlation, thus we further designed a bimolecular fluorescence complementation (BiFC) system of VN173-tagged TAPP1 PH domain and VC155-tagged ARAP3 PH4 domain (Fig. 2f, Supplementary S5). If TAPP1 PH and ARAP3 PH domains are close each other, the N- and C-terminal fragments of Venus (VN173 and VC155) can be reassembled to recover its yellow fluorescence^44^. Indeed, we can observe yellow fluorescence at vesicles, confirming strong interaction between ARAP3 PH4 domain and TAPP1 PH domain (Fig. 2g), probably mediated by their binding to PI(3,4)P_2_. We further confirmed that direct addition of PI(3,4)P_2_ can induce the yellow BiFC signal from VN173-ARAP3 PH4 and VC155-TAPP1 PH domain (Supplementary S6). Therefore, these results suggest that ARAP3 can be physically attracted to PI(3,4)P_2_-containing vesicles via its PH domains.

### PI(3,4)P2-enriched mature endosomes facilitates the degradation of ARAP3

**Figure 3:**
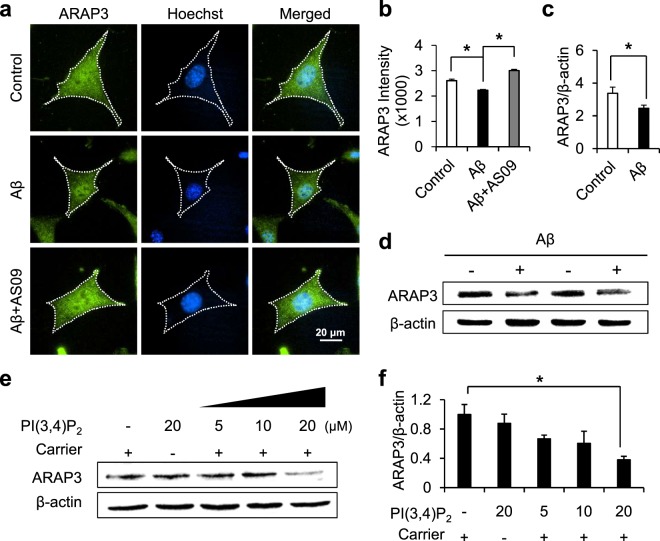
Aβ-mediated SHIP2 activation reduces the expression level of ARAP3. (**a**) Representative images of ARAP3 (green) in HT22 cells incubated without or with 1 μM Aβ in the absence or presence of 10 μM AS09. Cell nuclei were stained with hoechst33342 (blue). (**b**) The fluorescence intensity of cytosolic ARAP3 was quantified (means ± SEM; *t*-test; *p < 0.05; *n* = 78~100 cells per group). (**c**,**d**) The expression levels of ARAP3 in SH-SY5Y cells with or without the treatment of 1 μM Aβ for 24 hr. ARAP3 level was quantified by densitometry analysis and normalized to β-actin (means ± SEM; *t*-test; *p < 0.05; *n* = 4). (**e**) The expression level of ARAP3 in HT22 cells treated by various concentrations of PI(3,4)P_2_ with or without histone carriers for 24 hr. β-actin was used as a loading control. (**f**) ARAP3 level was quantified by densitometry analysis and normalized to β-actin (means ± SEM; *t*-test; *p < 0.05; *n* = 3).

We learned that Aβ-mediated SHIP2 activity induces the accumulation of PI(3,4)P_2_-enriched vesicles near perinuclear regions (Fig. 1). In addition, we found that ARAP3 can directly bind to PI(3,4)P_2_-positive vesicles via its PH domains (Fig. 2). Thus, we then tried to check the effect of Aβ treatment on ARAP3. Interestingly, in HT22 cells treated with Aβ for 24 hr, we observed that the level of cytosolic ARAP3 is significantly decreased (Fig. 3a,b). The Aβ-mediated decrease in the ARAP3 expression level was also confirmed by western blotting in SHSY5Y cells (Fig. 3c,d). This reduction of cytosolic ARAP3 level was prevented by SHIP2 inhibitor AS09 (Fig. 3a,b).

These results imply that Aβ-mediated SHIP2 activity and the subsequent accumulation of PI(3,4)P_2_-positive vesicles may contribute to the reduced level of ARAP3. To test whether the increase of PI(3,4)P_2_ can decrease the ARAP3 level, we next directly applied PI(3,4)P_2_ to cells. For the efficient intracellular delivery, PI(3,4)P_2_ was complexed with polyamine carrier histone H1^45^. Our results showed that, indeed, the ARAP3 level was significantly decreased by direct addition of 20 μM PI(3,4)P_2_ with the carrier (Fig. 3e,f), suggesting the overproduced PI(3,4)P_2_ contributes to the reduced level of ARAP3.

### Aβ-mediated PI(3,4)P2-positive vesicles are mature endosomes

We showed that Aβ induces the accumulation of PI(3,4)P_2_-positive vesicles, which can physically recruit ARAP3, and this facilitates the reduction of ARAP3 expression. We hypothesized that Aβ-induced endocytosis and the subsequent lysosomal pathway may be related to the decreased level of ARAP3. Thus, we next investigated the maturation stage of the PI(3,4)P_2_-containing vesicles at perinuclear regions.

**Figure 4:**
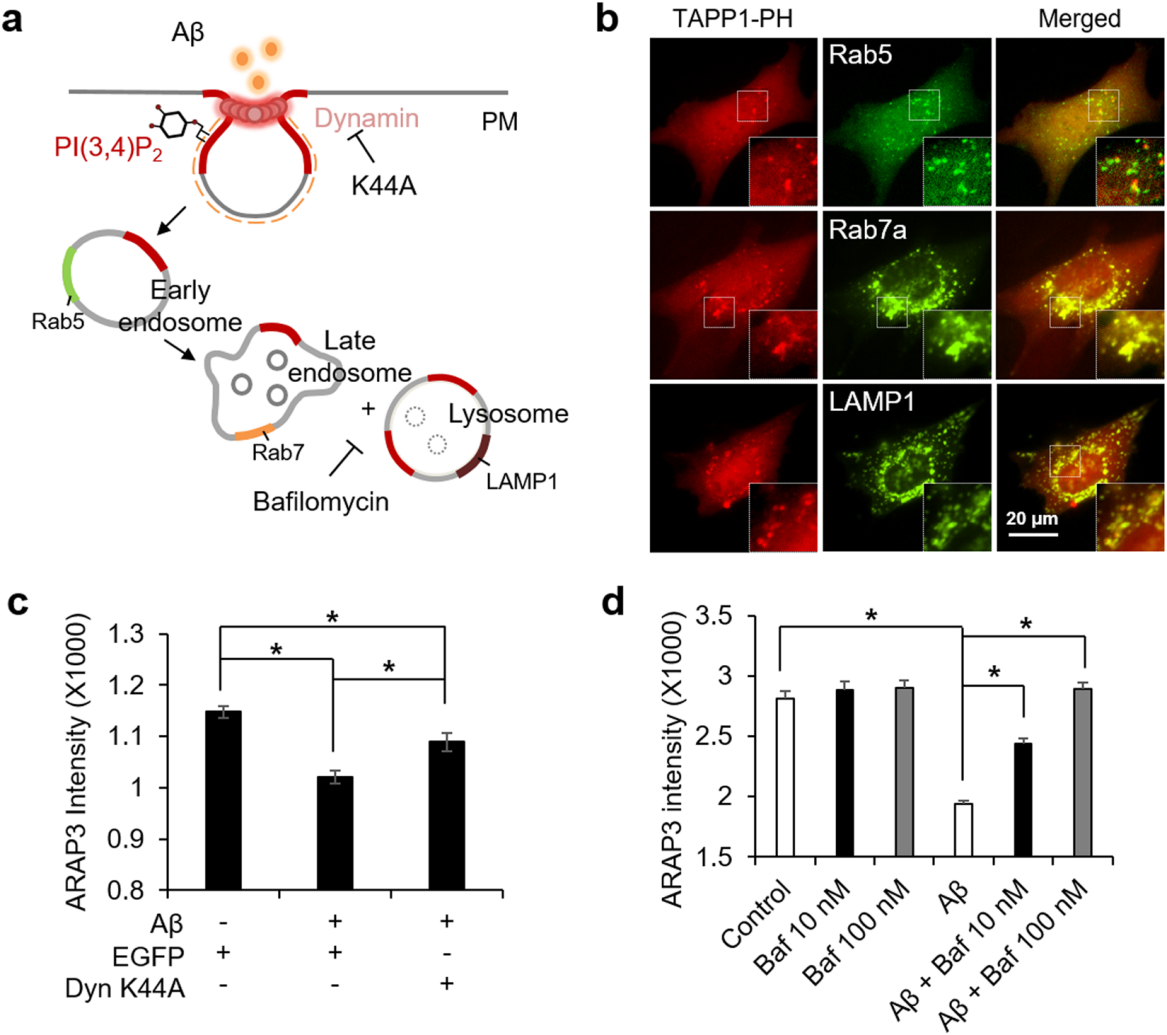
Aβ-mediated accumulation of PI(3,4)P2 facilitates endocytosis and lysosomal pathway of ARAP3. (**a**) Schematic representation of endosome maturation process. Different endosomal markers and inhibitors are displayed. (**b**) Distribution of different endosomal markers, Rab5, Rab7a, and LAMP-1 (green) and PI(3,4)P_2_ labelled with mKate-TAPP1-PH (red), in HT22 cells treated by 1 μM Aβ for 24 hr. Merged images are shown in the right panels. Insets show the boxed areas at high magnification. (**c**) The cytosolic expression level of ARAP3 in HT22 cells expressing EGFP or EGFP-dynamin K44A treated with 1 μM Aβ for 24 hr. (means ± SEM; *t*-test; *p < 0.05; *n* = 32~40 cells per group). (**d**) The cytosolic expression level of ARAP3 in HT22 cells treated with 1 μM Aβ for 24 hr in the absence or presence of bafilomycin A1. (means ± SEM; *t*-test; *p < 0.05; *n* = 48~68 cells per group).

Different species of phosphoinositide are involved in the maturation process of internalized vesicles^1,3^. In particular, it has been suggested that PI(3,4)P_2_ recruits the BAR proteins for the membrane constriction during initial stage of endocytosis, and then converted to PI3P in early endosomes^11,13,14^. Thus, the PI(3,4)P_2_-positive vesicles are expected to be found mainly in early endosomes. However, when we compared the distribution of the vesicles with different endosomal markers Rab5a, Rab7a and LAMP1, the PI(3,4)P_2_-enriched vesicles at perinuclear regions were surprisingly colocalized with Rab7a and LAMP1 which represent late endosomes and lysosomes (Fig. 4a,b).

In fact, PI(3,5)P_2_ has been known as the major phosphoinositide of late endosomes and lysosomes^1^, thus we further wondered whether the Aβ-induced accumulation of PI(3,4)P_2_ at perinuclear regions affects the distribution of PI(3,5)P_2_. Our result showed that, in the control cells, PI(3,5)P_2_ is mainly found at the vesicles near perinuclear regions, but this normal distribution of PI(3,5)P_2_ at perinuclear regions was significantly reduced by the treatment of Aβ (Supplementary Fig. S7). In addition, segmented distribution of PI(3,5)P_2_ at plasma membrane was observed in the Aβ-treated cells (Supplementary Fig. S7a). Therefore, our results suggest that Aβ-induced and SHIP2-mediated overproduction of PI(3,4)P_2_ and its abnormal accumulation at mature endosomes near perinuclear regions further alter normal distribution of other phosphoinositide PI(3,5)P_2_.

### Aβ-mediated lysosomal pathway reduced the level of ARAP3

We observed the Aβ-induced alteration of phosphoinositides, in particular the accumulation of PI(3,4)P_2_ at mature endosomes. This can be related to the facilitation of lysosomal pathway of ARAP3. To test this hypothesis, we first introduced dominant negative mutant of dynamin DynK44A, which is essential for membrane fission during endocytosis. The reduction of the ARAP3 level by Aβ was indeed prevented when dynamin mutant K44A blocks the completion of the internalization of endocytic vesicles (Fig. 4c, Supplementary Fig. S8a), suggesting that the Aβ-induced endocytosis is important for the decreased ARAP3 level. Furthermore, we applied a selective inhibitor of vacuolar H^+^ ATPase, bafilomycin A1, which blocks acidification and protein degradation in lysosomes. The results showed that the Aβ-induced decrease in ARAP3 level was completely prevented by the treatment of 100 nM bafilomycin (Fig. 4d, Supplementary Fig. S8b). These results suggest that the reduced ARAP3 level is due to the protein degradation through Aβ-induced endocytosis and the following lysosomal pathway.

### Decreased ARAP3 level causes RhoA hyperactivation and F-actin formation

**Figure 5:**
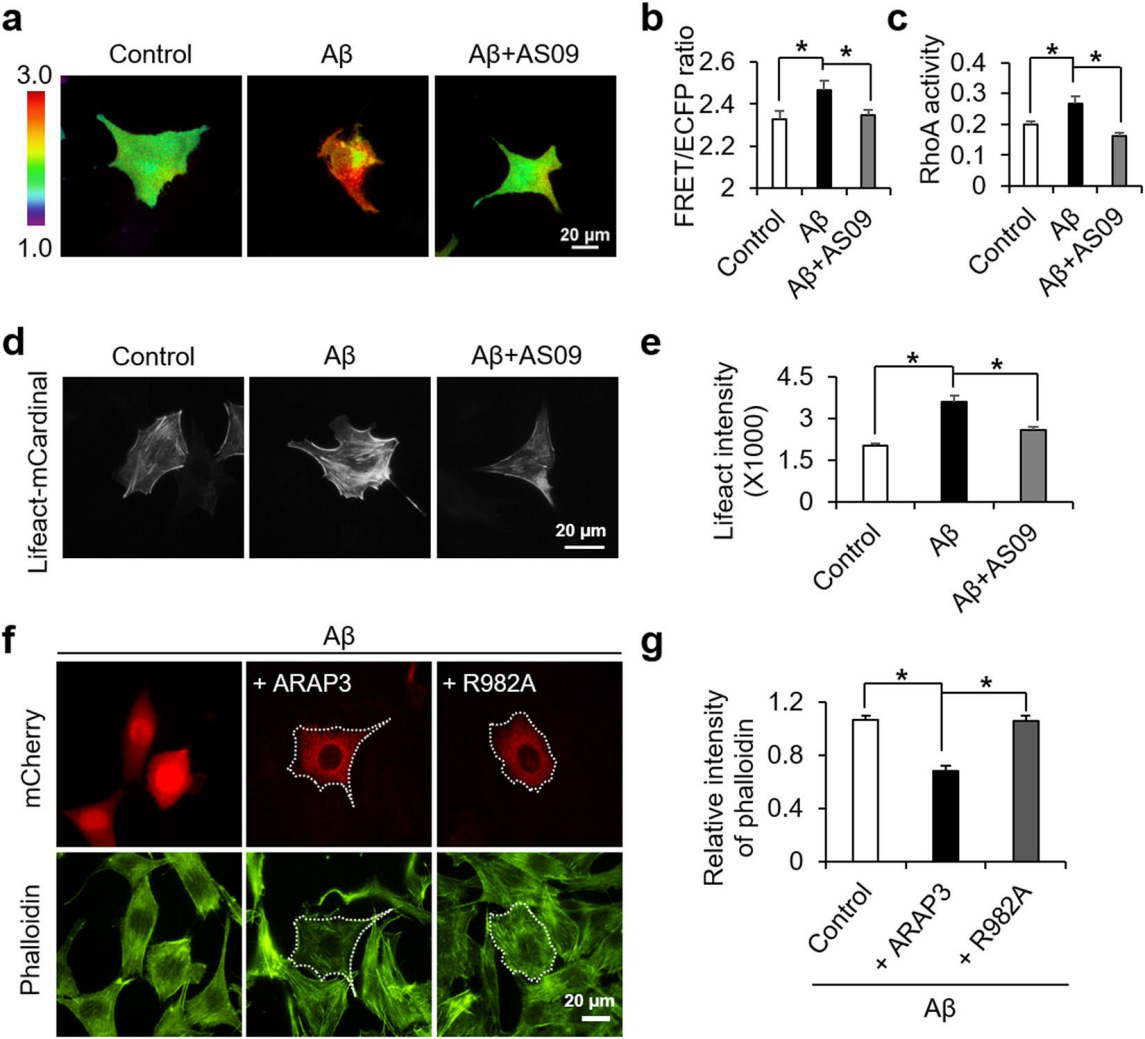
Aβ-induced RhoA hyperactivation and the accumulation of F-actin are dependent on SHIP2 activity. (**a**,**b**) Representative images (**a**) and the averaged values (**b**) of the FRET/CFP emission ratio images of RhoA biosensor in the HT22 cells stimulated with 1 μM of Aβ in the absence or presence of 10 μM AS09 for 24 hr (means ± SEM; *t*-test; *p < 0.05; *n* = 40~42 cells per group). (**c**) Active RhoA was quantified using ELISA-based RhoA activation assay (means ± SEM; *t*-test; *p < 0.05; *n* = 4). (**d**,**e**) Representative images (**d**) and the quantified intensity (**e**) of lifeact-mCardinal in HT22 cells treated with 1 μM Aβ in the absence or presence of 10 μM AS09 for 24 hr (means ± SEM; *t*-test; *p < 0.05; *n* = 80~88 cells per group). (**f**,**g**) Representative images (**f**) and the quantification analysis (**g**) of phalloidin after the stimulation with 1 μM of Aβ for 24 hr in HT22 cells overexpressing ARAP3 or R982A mutant (means ± SEM; *t*-test; *p < 0.05; *n* = 32~40 cells per group).

To investigate the downstream effect of the Aβ-mediated decrease in ARAP3 level, we utilized RhoA biosensor based on fluorescence resonance energy transfer (FRET)^39^. Live-cell imaging with this RhoA FRET biosensor showed that RhoA activity was significantly increased by oligomeric Aβ (Fig. 5a,b), possibly by the reduced level of its negative regulator, ARAP3. In fact, this RhoA hyperactivation can be prevented by AS09 (Fig. 5a,b), which inhibits the SHIP2-mediated decrease in ARAP3 level (Fig. 3a,b). These results were further confirmed by ELISA assay for RhoA activity (Fig. 5c).

RhoA is a key regulator of actin dynamics by formation of filomentous actin (F-actin) structures^46^. Also, pathological actin in a polymerized conformation has been reported in several neurodegenerative disorders including Alzheimer’s disease^41,47^. Thus, we evaluated the degree of actin polymerization using mCardinal-tagged Lifeact, which allows detection of actin dynamics in live cells^48^. Our results showed that the level of actin polymerization was dramatically increased upon Aβ stimulation, and its enhancement was inhibited by the SHIP2 inhibitor AS09 (Fig. 5d,e).

**Figure 6:**
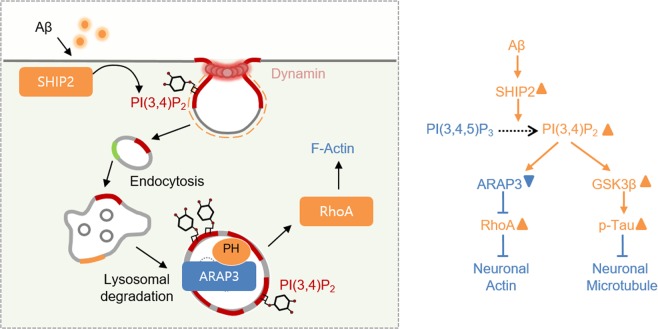
A model summarizing the Aβ-induced actin cytoskeletal abnormalities via SHIP2-PI(3,4)P_2_-ARAP3-RhoA signaling pathway.

To further confirm that this is due to SHIP2-mediated reduction in ARAP3 level, we introduced wild type ARAP3 or its negative mutant R982A to the HT22 cells treated with Aβ. Our results clearly showed that the Aβ-induced increase in F-actin was prevented by overexpression ARAP3, but not its mutant R982A (Fig. 5f,g). These results suggest that Aβ can induce the SHIP2-mediated accumulation of PI(3,4)P_2_-positive vesicles at mature endosomes, which reduces the level of ARAP3, mediating RhoA hyperactivation and F-actin formation (Fig. 6).

## Discussion

Abnormality in neuronal cytoskeleton is critical for neurotoxicity and synaptic dysfunction in Alzheimer’s disease^49^. For example, neuronal microtubule is a major cytoskeleton composing axonal structure and thus pathological tau aggregation causes severe neurotoxicity in AD^50^. Neuronal actin is crucial for neurite dynamics and synaptic functions, and pathological accumulation of filamentous actin can result in synaptic dysfunctions in AD^51^. Therefore, underlying molecular mechanisms in the Aβ-induced disruption of neuronal cytoskeleton have been heavily investigated^49,52^. Particularly, a recent study reported the molecular links between Aβ and tau phosphorylation through FcγRIIb-mediated SHIP2 activation^21^.

Our current results provide important molecular links between Aβ and actin disruption, which can potentially influence synaptic dysfunctions in Alzheimer’s disease. Previous reports showed that Aβ pathology is associated with increased accumulation of F-actin via Rho GTPases^36^. We also confirmed that Aβ induces hyperactivation of RhoA, and further discovered that it can be due to the decreased expression level of a negative RhoA regulator ARAP3. Surprisingly, the reduction of ARAP3 was facilitated by elevated SHIP2 activity and its phospholipid product PI(3,4)P_2_. In particular, this over-produced PI(3,4)P_2_ was accumulated at the endocytic vesicles involved in the lysosomal pathway, leading to the reduction of ARAP3 level. Thus, our results provide new molecular mechanisms underlying the Aβ-mediated pathological actin polymerization.

Our results suggest that Aβ-induced actin disruption can be mediated by a lipid phosphatase SHIP2 and dysregulated phosphoinositide metabolism. Similar to our current results, it has been shown that Aβ influences the metabolism of PI(4,5)P_2_ by altering another lipid phosphatase synaptojanin^19,20^. In our study, the Aβ-induced and SHIP2-mediated production of PI(3,4)P_2_ caused the increase of endocytic vesicles (Fig. 1). This pathologically over-produced PI(3,4)P_2_ is surprisingly localized at late endosomes and lysosomal vesicles (Fig. 4b), while PI(3,4)P_2_ has been suggested to play a crucial role in the early stage of endocytosis^3,8,11,15^. Furthermore, this PI(3,4)P_2_-positive endosomes can physically interact with the PH domain of ARAP3 (Fig. 2) and this facilitates the degradation process of ARAP3 (Fig. 3). This is the first evidence that the accumulation of PI(3,4)P_2_ at mature endosomes can induce the degradation of ARAP3, which is crucial for the integrity and dynamics of actin cytoskeleton. These results are interesting because it has been generally known that the major lipid component of late endosomes and lysosomes is PI(3,5)P_2_^16^. We further confirmed that the distribution of PI(3,5)P_2_ is also altered in the Aβ-treated cells (Fig. S7). Therefore, our results suggest that Aβ-induced dysregulation of phosphoinositide metabolism can alter the phosphoinositide component of endosomal vesicles at different stages.

ARAP3 is an interesting molecule known to interact with both phosphoinositides and their metabolizing enzyme SHIP2^28^. In addition to direct interaction with SHIP2, ARAP3 can be present in a multimeric complex with SHIP2 and a Cbl-interacting protein CIN85^28,53^, which is important for receptor endocytosis^54^. ARAP3 also contains 5 PH domains, which is a well-known domain for phosphoinositide binding, and it has been shown that the N-terminal PH1 and PH2 domains bind to PI(3,4,5)P_3_, recruiting ARAP3 to plasma membrane^35^. Importantly, we further discovered that PH4 domains of ARAP3 may be crucial for its association to PI(3,4)P_2_ (Fig. 2), which can physically bring ARAP3 to the PI(3,4)P_2_-mediated lysosomal pathway.

An intriguing question is how PI(3,4)P_2_ is accumulated in the lysosomal endosomes. One possible mechanism is that over-produced PI(3,4)P_2_ makes it difficult to stay only in the early endosomes, allowing its distribution in the other stages of vesicles such as late endosomes. Another possible mechanism is that the conversion of PI(3,4)P_2_ to PI3P in the early endosome and/or PI3P to PI(3,5)P_2_ in the lysosomal endosomes are further unbalanced by Aβ. In fact, we observed altered distribution of PI(3,5)P_2_ (Fig. S7). Also, a recent study demonstrated that PI3P is deficient in the brain tissue from AD patients and AD mouse models^55^. These evidences imply more complex pathological mechanisms for the alteration of multiple phosphoinositide metabolism in AD. While we focused on the SHIP2-mediated over-production of PI(3,4)P_2_ and its effect on actin disruption in the current study, further systematic investigations utilizing advanced fluorescent biosensors will be needed to understand complex phospholipid metabolism altered in Alzheimer’s disease.

Taken together, we discovered new molecular mechanisms on how Aβ can cause the disruption of actin cytoskeleton through dysregulated phosphoinositide metabolism in Alzheimer’s disease. We observed that the Aβ-induced accumulation of PI(3,4)P_2_ at mature endosomes facilitates the degradation of ARAP3, which causes actin disruption via RhoA hyperactivation. It is interesting that the Aβ-induced alteration of SHIP2 function has been previously shown to mediate tau phosphorylation as well. Because SHIP2 plays a central role in the Aβ-induced abnormality of both neuronal actin and microtubule, it can be considered as a powerful therapeutic target for Alzheimer’s disease. In our study, we utilized fluorescent biosensors which can specifically monitor dynamic molecular events in live cells with high spatiotemporal resolutions. These advanced fluorescent biosensors and live-cell imaging technology will promise further discovery of dynamic molecular and pathological mechanisms of phospholipid metabolism.

## Methods

### Plasmids

To construct the mKate2-TAPP1-PH, BamHI-mKate2 and TAPP1-PH-Not1 were prepared by PCR with mKate2-P2A-APEX2-TAPP1-PH (Addgene number; #67662) as a template. These PCR products were inserted in pRK5 vector using In-Fusion technique (Takara). pTag-BFP-C-h-Rab5a-c-Myc (#79801), GFP-Rab7a (#61803), LAMP1-mGFP (#34831), and pEGFPN1-human Dynamin K44A (#22197) were obtained from Addgene. FRET-based RhoA biosensor is originally from Dr. Matsuda (Osaka University)^39^. mCherry-tagged ARAP3 and ARAP3-R982A were generated by fusing red fluorescent protein mCherry into the N-terminal of the ARAP3 templates^34^. To construct the EGFP-ARAP3-PH(X), each PH domain^35^ and EGFP sequences were prepared by PCR and inserted in pRK5 vector using In-Fusion technique. VN173-tagged TAPP1 was constructed by inserting PCR fragments of VN173 and TAPP1 PH domain in pRk5 vector. VC155-tagged ARAP3-PH(X) constructs were generated by *Xba1/BamH1* replacement of EGFP in EGFP-ARAP3-PH(X) with a VC155 including *Xba1/BamH1* site.

### Reagents

PI(3,4)P_2_-diC16 (#P3416) and histone H1 carrier (#P-9C2) were purchased from Echelon. Bafilomycin A1 was obtained from Sigma-Aldrich. Alexa 488 Phalloidin and Hoechst33342 were purchased from Thermo Fisher Scientific. SHIP2 siRNA was purchased from Bioneer (#16333-1 and 16333-2).

### Preparation of amyloid β oligomers

Aβ_1-42_ peptide (Bachem) was prepared as previously described^42^. Briefly, the lyophilized peptide was dissolved in ice-cold 1,1,1,3,3,3-Hexafluoro-2-Propanol (HFIP, Sigma-Aldrich) and incubated for 2 hr at room temperature to allow for Aβ monomerization. HFIP was evaporated in a fume hood until a clear peptide film is observed. The peptide film can be incubated at −80 °C until use. Aβ stock solution was prepared from this peptide film by adding DMSO to a final concentration of 5 mM. For the experiment, Aβ_1–42_ stock solution was further diluted to 100 μM in PBS and incubated overnight at 4 °C to achieve oligomeric Aβ solution.

### Cell culture

Cells were maintained in DMEM supplemented with 10% fetal bovine serum (Hyclone), 1 unit per ml penicillin, 100 μg per ml streptomycin. Cell culture reagents were purchased from Hyclone. Cells were cultured in a humidified 95% air, 5% CO_2_ incubator at 37 °C. The cells were transiently transfected with indicated constructs by Lipofectamine^TM^ 2000 reagent (Invitrogen) according to the manufacture’s protocol. Following incubation, the cells were maintained in neurobasal media (Gibco) containing B27 (Gibco), L-glutamine (Invitrogen) and penicillin/streptomycin, and then treated with 1 μM of oligomeric Aβ_1–42_ for 24 hr.

### Cell viability assay

The EZ-cytox assay kit (Daeillab) was used to measure the cytotoxicity of Aβ. Briefly, cells were seeded in 96-well plate at a density of 5 × 10^4^ cells/ml in a volume of 100 μl/well. Following 24 hr of incubation, the cells were maintained in neurobasal media (Gibco) containing B27 (Gibco), L-glutamine (Invitrogen) and penicillin/streptomycin, and then treated with 1 μM of oligomeric Aβ_1–42_ for 24 hr. After incubation, 10 μl of WST reagent solution (water-soluble tetrazolium salt) was added to each well and plates were incubated for 1 hr at 37 °C. The absorbance of living cells was revealed at 450 nm using a microplate reader. The percentage of living cells was calculated in comparison to the control cells.

### Image acquisition

For the fluorescence imaging, cells were prepared on cover-glass-bottom dishes (SPL Life Sciences) coated with 10 μg/ml of fibronectin (Invitrogen). FRET images were collected by a Nikon Ti-E inverted microscope and a cooled charge-coupled device camera using NIS-Elements software (Nikon) with a 438DF24 excitation filter, a 458DRLP dichroic mirror, and two emission filters controlled by a filter changer (483DF32 for SECFP and 542DF27 for Venus). Red fluorescent images were collected using a 562DF40 excitation filter, a 593DRLP dichroic mirror, and a 641DF75 emission filter. Blue fluorescent images were acquired using a 377DF50 excitation filter, a 409 dichroic mirror, and a 447DF60 emission filter. Green fluorescent images and Venus images were collected using a 482DF35 excitation filter, a 506DRLP dichroic mirror, and a 536DF40 emission filter. A neutral-density filter was used to control the intensity of the excitation light. The fluorescence intensity of non-transfected cells was quantified as the background signals and subtracted from the SECFP and Venus signals on transfected cells. The pixel-by-pixel ratio images of FRET/SECFP were calculated based on the background-subtracted fluorescence intensity images of SECFP and Venus by the NIS-Elements program to allow the quantification and statistical analysis of FRET responses.

### Immunostaining

After treatment of Aβ, cells were fixed with 4% paraformaldehyde for 20 min, and permeabilized with 0.1% Triton X-100 for 15 min. Cells were incubated in 5% BSA in PBS for 1 hr, and then incubated with rabbit anti-ARAP3 antibody (2 μg/ml, Atlas antibodies) or rabbit anti-PI(3,4)P_2_ antibody (1 μg/ml, Echelon) for overnight at 4 °C. After washing with PBS, cells were incubated with Alex-Fluor 488 or 594 goat anti-rabbit antibody (diluted 1: 200, Thermo Fisher Scientific) for 2 hours at room temperature in the dark. After another washing three times with PBS for 10 min each, samples were exposed to Hoechst33342 (1 μg/ml, Thermo Fisher Scientific) for 5 min. The stained cells were observed under a fluorescence microscopy.

### Western blotting

The protein was subjected to SDS-PAGE and blotted with ARAP3 antibody (1 μg/ml, Atlas antibodies). The equal amount of protein loading was detected and normalized with β-actin (Santa Cruz Biotechnology) on the same membrane. We developed western blot membranes with enhanced chemiluminescence (ECL) solution and images were captured with a luminescent image analyzer ChemiDoc (Bio-Rad). The quantification of band intensity on the blot was analyzed with a software program, Image Lab 5.2.1 (Bio-Rad).

### Intracellular delivery of PI(3,4)P2

The synthetic PI(3,4)P_2_-diC16-histone carrier complex (Echelon) was formed by incubating at room temperature for 15 min. The complex was diluted with neurobasal media and then added to the culture dishes. After 24 hr, cells were harvested in lysis buffer for western blotting.

### RhoA ELISA activation assay

The ELISA-based RhoA activity assay (#BK124; Cytoskeleton) was used to measure the GTP-RhoA, according to the manufacturer’s instructions. Briefly, HT22 cells were harvested in lysis buffer, immediately snap frozen in liquid nitrogen and stored at −70 °C to avoid GTP hydrolysis. Equal amounts of proteins were added to the wells coated with RhoA GTP binding protein, and the plates were incubated on an orbital microplate shaker at 4 °C for 30 min. After washing, the strips were incubated with an anti-RhoA primary antibody for 45 min. After incubation, the strips were incubated with HRP-conjugated secondary antibody for 45 min followed by HRP detection reagent. The absorbance was revealed at 490 nm using a microplate reader. The activity of RhoA was averaged and normalized to the control samples.

### Statistical analysis

P values were calculated by two-tailed Student’s t-test, and p < 0.05 was considered significant.

## Supplementary information

Supplementary Information

Supplementary Video 1
