# Supplementary material for: Aβ modulates actin cytoskeleton via SHIP2-mediated phosphoinositide metabolism
Source: Sci Rep. 2019 Oct 29;9:15557. doi: 10.1038/s41598-019-51914-2 (PMC6820556; doi:10.1038/s41598-019-51914-2)
Supplement: Supplementary file 1 — Supplementary Information [file 41598_2019_51914_MOESM1_ESM.docx]

Supplementary Information

**Aβ modulates actin cytoskeleton via SHIP2-mediated phosphoinositide metabolism**

Hae Nim Lee, Kyoung Mi Sim, Hyunbin Kim, Jeongmin Ju, Ae Nim Pae, Jae-Bong Park, Hoon Ryu, Jihye Seong*

**
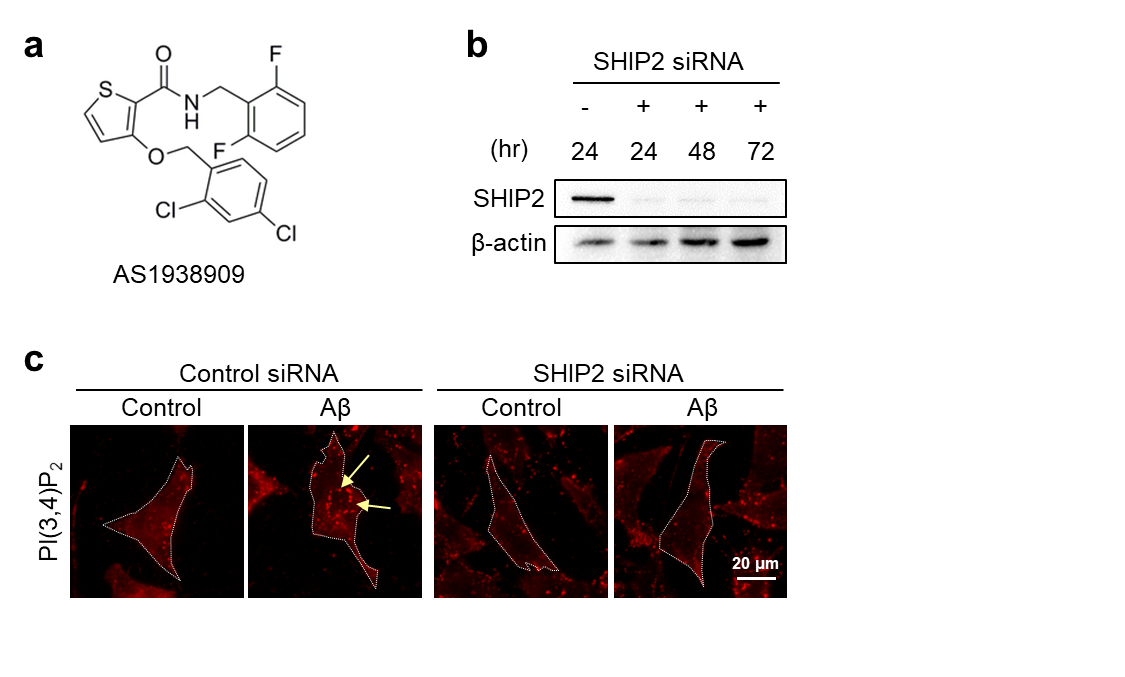
**

**Supplementary Figure S1.** *SHIP2 inhibition prevents the Aβ-induced accumulation of PI(3,4)P_2_ near perinuclear regions*

**(a)** SHIP2 inhibitor AS1938909. **(b)** Representative immunostaining images of PI(3,4)P*_2_* in HT22 cells expressing control siRNA or SHIP2 siRNA, with or without the treatment of Aβ for 24 hr. **(c**) The expression level of SHIP2 in HT22 cells at indicated times after transfection of SHIP2 siRNA (Bioneer, 16333-1 and 16333-2, 50 nM each). Actin was used as a loading control.


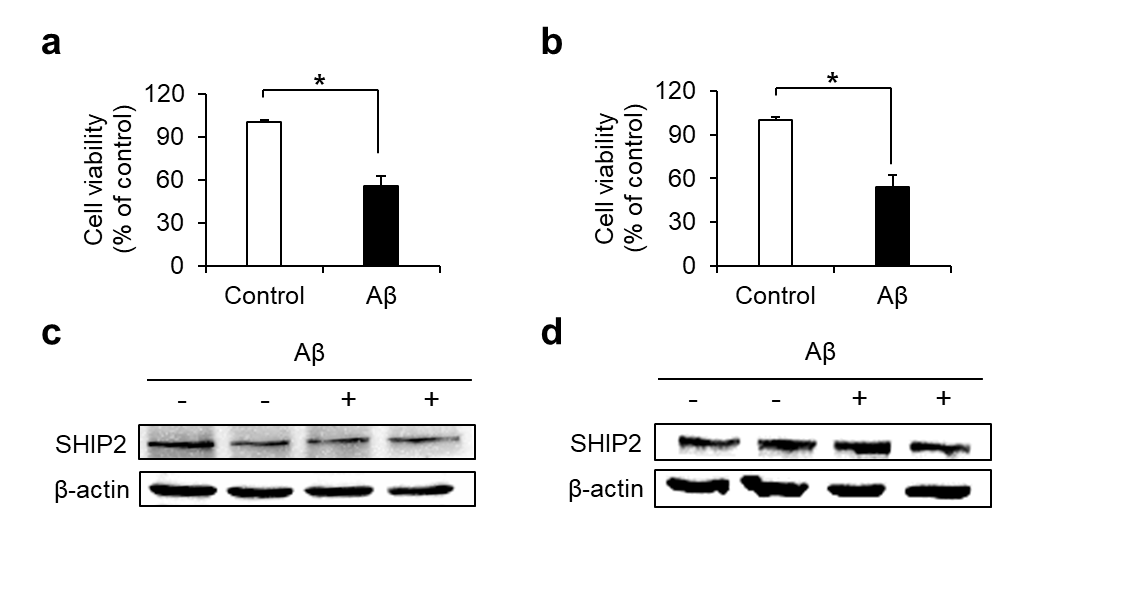


**Supplementary Figure S2.** *Aβ does not affect the SHIP2 expression level.*

**(a)** Cell viability of HT22 cells after the incubation of 1 μM oligomeric Aβ for 24 hr (means ± SEM; *t*-test; **p* < 0.05; *n* = 5 cells per group). **(b)** Cell viability of SH-SY5Y cells after the incubation of 1 μM oligomeric Aβ for 24 hr (means ± SEM; *t*-test; **p* < 0.05; *n* =4 cells per group). **(c-d)** The expression levels of SHIP2 in HT22 **(c)** or SH-SY5Y cells **(d)** treated with or without 1 μM oligomeric Aβ for 24 hr. Actin was used as a loading control.

**
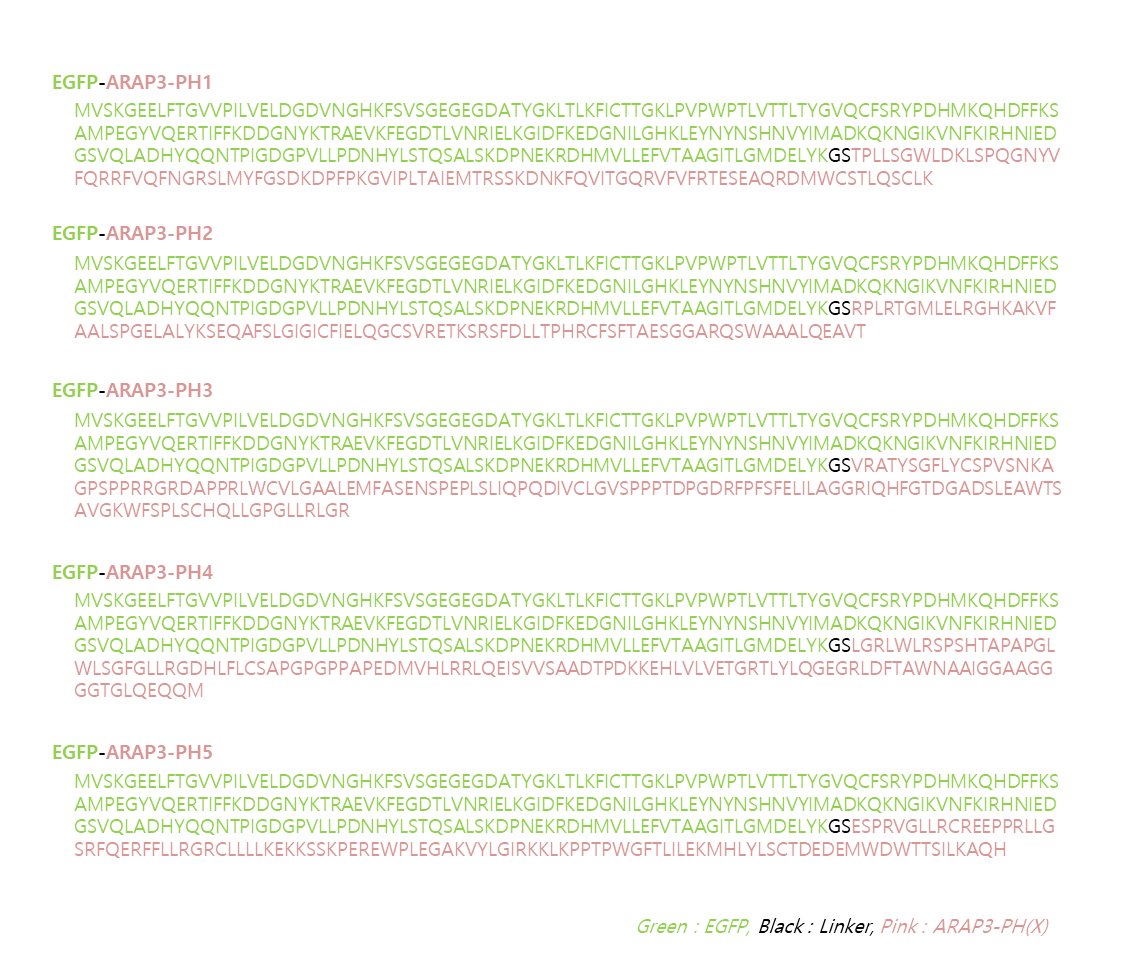
 Supplementary Figure S3.** *Schematic diagram of EGFP-ARAP3-PH domains*

Amino acid sequences of EGFP-ARAP3-PH1, PH2, PH3, PH4 and PH5 (Green; EGFP, Black; Linker, Pink; ARAP3-PH1 to PH5).

**
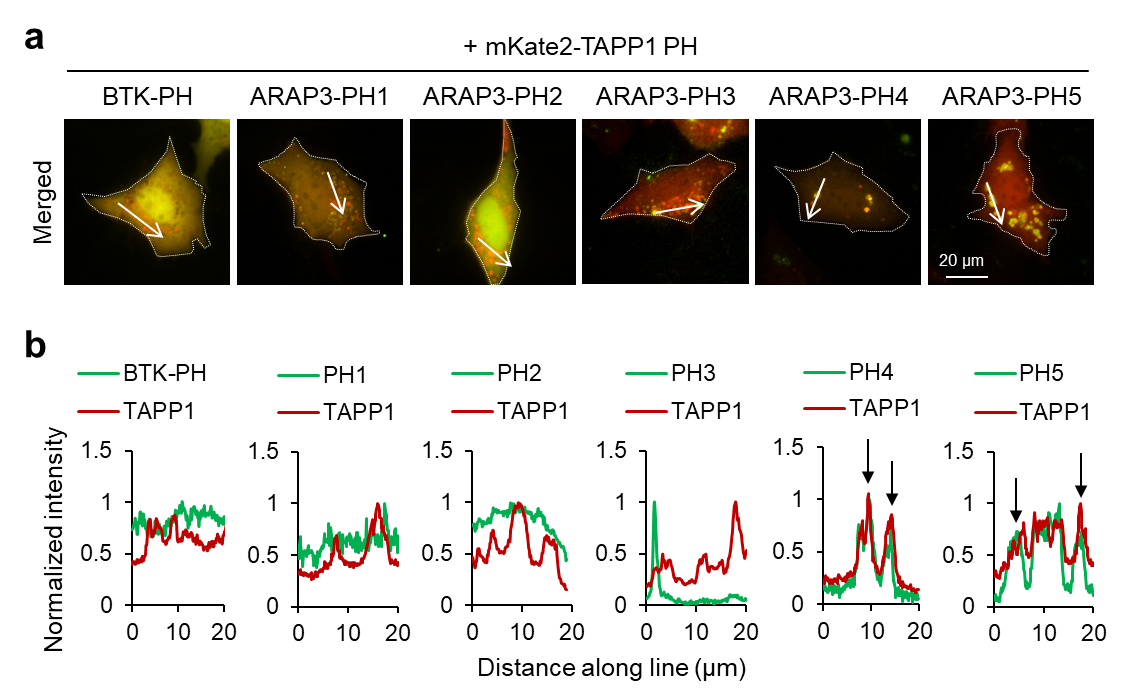
**

**Supplementary Figure S4.** *ARAP3 can be associated with PI(3,4)P_2_-containing vesicles via its PH4 and PH5 domains.*

**(a)** Representative merged images of EGFP-tagged each PH domain of ARAP3 (green) and mKate2-TAPP1 (red) in HT22 cells after 48hr starvation. Btk PH domain-EGFP was used as control. **(b)** Intensity profiles were acquired along the indicated lines in the merged image shown in **(a)**.**
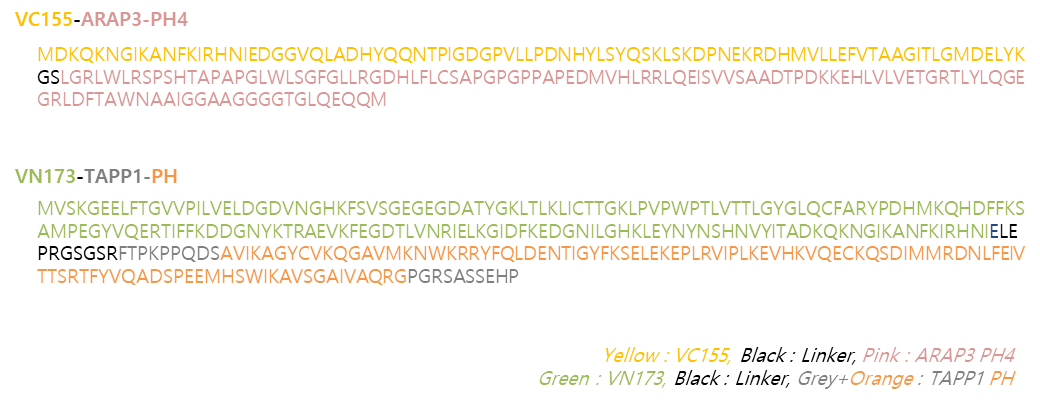
 Supplementary Figure S5.** *BiFC system of VC155-ARAP3-PH4 and VN173-TAPP1-PH*

Amino acid sequences of VC155-ARAP3-PH1, PH4 and VN173-TAPP1-PH (Orange; VC155, Blue; VN173, Black; Linker, Red; ARAP3-PH(X), Gray; TAPP1-PH).

**
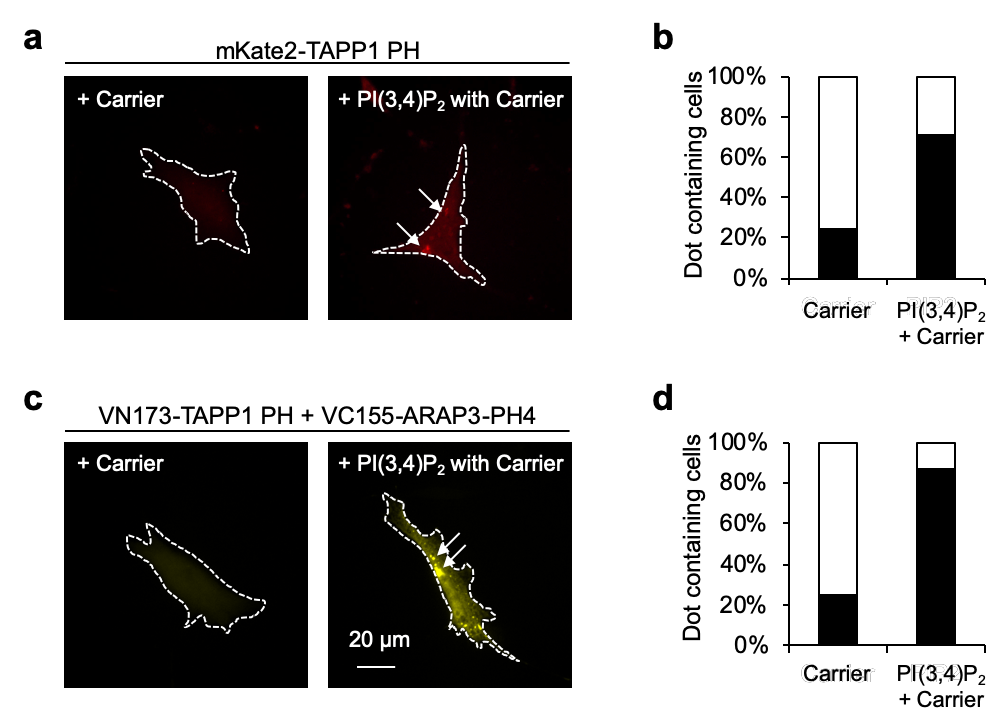
Supplementary Figure S6.** *Direct addition of PI(3,4)P_2_ complexed with histone carrier induces yellow BiFC signal from VC155-ARAP3-PH4 and VN173-TAPP1-PH.*

(**a**-**b**) Representative images of mKate2-TAPP1 PH domain treated by histone carrier without or with 20 μM PI(3,4)P_2_ for 1 h (**a**). The distribution of PI(3,4)P_2_ was observed as red dots (arrows). The cells treated with PI(3,4)P_2_ show significantly higher rate of red dots (**b**). (Fisher’s exact test; *p* < 0.05; *n* =58 and 98). (**c**-**d**) Representative images of BiFC system treated by histone carrier without or with 20 μM PI(3,4)P_2_ for 1 h (**c**). The distribution of PI(3,4)P_2_ was observed at yellow dots (arrows). The cells treated with PI(3,4)P_2_ show significantly higher rate of yellow dots (**d**). (Fisher’s exact test; *p* < 0.05; *n* =63 and 62).

**
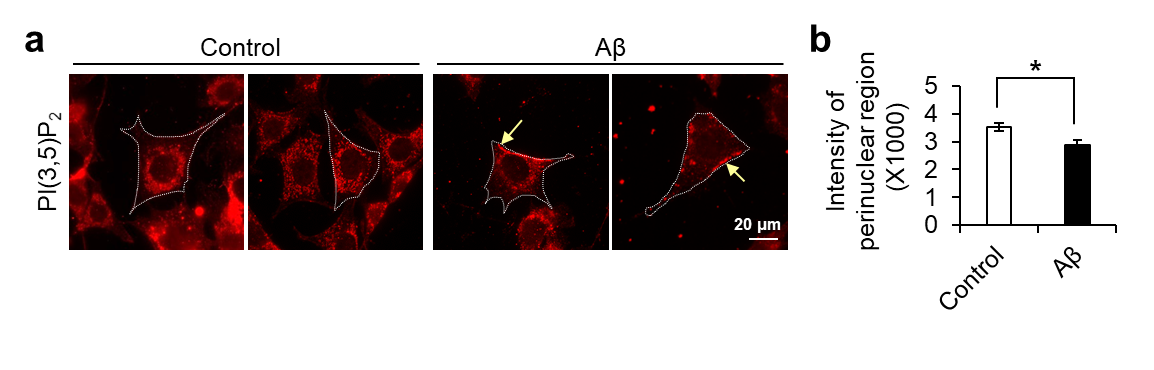
Supplementary Figure S7.** Aβ-induced alteration of PI(3,5)P*_2_* distribution in HT22 cells

**(a**) Representative immunostaining images of PI(3,5)P*_2_* in HT22 cells with or without the treatment of 1 μM Aβ for 24 hr. **(b)** The average fluorescence intensity in the perinuclear regions in cells with or without the treatment of 1 μM Aβ for 24 hr (means ± SEM; *t*-test; **p* < 0.05; *n* = 41~42 cells per group).

**
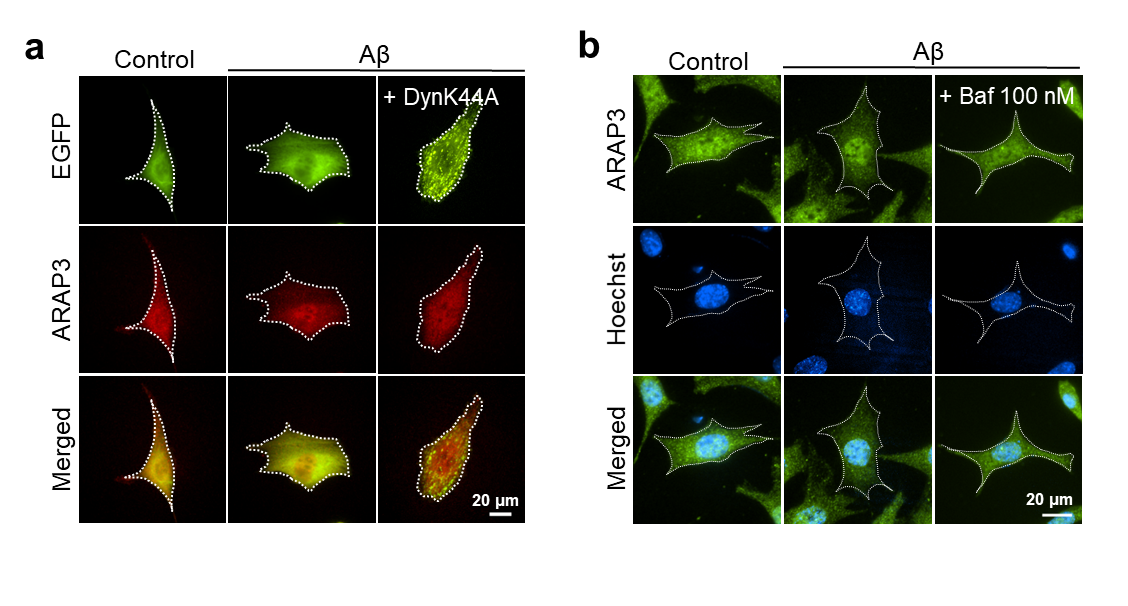
**

**Supplementary Figure S8.** *Aβ-mediated accumulation of PI(3,4)P_2_ facilitates endocytosis and lysosomal pathway of ARAP3.*

**(a)** Representative images of ARAP3 expression level (red) in HT22 cells expressing EGFP or EGFP-dynamin K44A (green) treated with 1 μM Aβ for 24 hr. Merged images are shown in the lower panels. The quantification result is shown in Fig. 4c. **(b)** Representative images of ARAP3 levels (green) in HT22 cells treated with 1 μM Aβ for 24 hr in the absence or presence of bafilomycin A1. Cell nuclei were stained with Hoechst (blue). The quantification result is shown in Fig. 4d.

**Supplementary Movie Legend**

**Supplementary Movie 1.** *Aβ-induced real-time changes in the distribution of TAPP1-PH-mKate in HT22 cells.*
